# Contractile Force Is Enhanced in Aortas from Pendrin Null Mice Due to Stimulation of Angiotensin II-Dependent Signaling

**DOI:** 10.1371/journal.pone.0105101

**Published:** 2014-08-22

**Authors:** Roy L. Sutliff, Erik R. Walp, Young Hee Kim, Lori A. Walker, Alexander M. El-Ali, Jing Ma, Robert Bonsall, Semra Ramosevac, Douglas C. Eaton, Jill W. Verlander, Laura Hansen, Rudolph L. Jr. Gleason, Truyen D. Pham, Seongun Hong, Vladimir Pech, Susan M. Wall

**Affiliations:** 1 Atlanta Veterans Affairs Medical Center, Atlanta, Georgia, United States of America; 2 Department of Medicine, Emory University, Atlanta, Georgia, United States of America; 3 Departments of Medicine and Cardiology, University of Colorado Health Sciences Center, Aurora, Colorado, United States of America; 4 Department of Psychiatry and Behavioral Sciences, Emory University, Atlanta, Georgia, United States of America; 5 Department of Physiology, Emory University, Atlanta, Georgia, United States of America; 6 Department of Medicine, University of Florida, Gainesville, Florida, United States of America; 7 School of Mechanical Engineering, Georgia Institute of Technology, Atlanta, Georgia, United States of America; Institut National de la Santé et de la Recherche Médicale, France

## Abstract

Pendrin is a Cl^−^/HCO_3_^−^ exchanger expressed in the apical regions of renal intercalated cells. Following pendrin gene ablation, blood pressure falls, in part, from reduced renal NaCl absorption. We asked if pendrin is expressed in vascular tissue and if the lower blood pressure observed in pendrin null mice is accompanied by reduced vascular reactivity. Thus, the contractile responses to KCl and phenylephrine (PE) were examined in isometrically mounted thoracic aortas from wild-type and pendrin null mice. Although pendrin expression was not detected in the aorta, pendrin gene ablation changed contractile protein abundance and increased the maximal contractile response to PE when normalized to cross sectional area (CSA). However, the contractile sensitivity to this agent was unchanged. The increase in contractile force/cross sectional area observed in pendrin null mice was due to reduced cross sectional area of the aorta and not from increased contractile force per vessel. The pendrin-dependent increase in maximal contractile response was endothelium- and nitric oxide-independent and did not occur from changes in Ca^2+^ sensitivity or chronic changes in catecholamine production. However, application of 100 nM angiotensin II increased force/CSA more in aortas from pendrin null than from wild type mice. Moreover, angiotensin type 1 receptor inhibitor (candesartan) treatment in vivo eliminated the pendrin-dependent changes contractile protein abundance and changes in the contractile force/cross sectional area in response to PE. In conclusion, pendrin gene ablation increases aorta contractile force per cross sectional area in response to angiotensin II and PE due to stimulation of angiotensin type 1 receptor-dependent signaling. The angiotensin type 1 receptor-dependent increase in vascular reactivity may mitigate the fall in blood pressure observed with pendrin gene ablation.

## Introduction

Pendrin is an electroneutral Cl^−^/HCO_3_^−^ exchanger expressed in the apical regions of a minority cell type that localizes to the cortical collecting duct and connecting tubule in the kidney, where it mediates absorption of Cl^−^ and secretion of HCO_3_^−^ [1]–[4]. Aldosterone and angiotensin II greatly stimulate pendrin abundance and function, thereby increasing renal Cl^−^ absorption, which contributes to the hypertension observed following the administration of these hormones [5]–[7]. Disruption of the gene encoding pendrin (*Slc26a4*) augments renal NaCl excretion [8], [9], which blunts the increase in blood pressure observed with aldosterone administration [5], [9]. Therefore, pendrin modulates blood pressure, at least in part, by mediating renal Cl^−^ absorption, which expands vascular volume.

Hypertension is accompanied by changes in vascular reactivity, which occurs through both structural changes in the walls of blood vessels [10], [11] and through changes in the sensitivity of blood vessels to vasoconstrictors [12], [13]. In many models of hypertension, such as in senescent, spontaneously hypertensive rats [14] or in angiotensin II-treated mice [15], increased vascular reactivity and hypertrophy are observed. Since blood pressure is lower in pendrin null than in wild type mice [9], [16], reduced vascular tone is expected. However, pendrin null mice have elevated plasma renin concentration [8], [9], which should increase plasma angiotensin II concentration, thereby stimulating vascular contractility [15]. To resolve these issues, the effect of pendrin ablation on vascular reactivity was examined in aortic rings. The purpose of this study was threefold: 1) to determine if pendrin is expressed in mouse aorta, 2) to determine if the lower blood pressure observed in pendrin null mice occurs in tandem with reduced vascular contractile function and 3) to ascertain the mechanism by which this occurs.

## Methods

### Animals

Pendrin null (*Slc26a4* (−/−)) mice developed by Everett et al [17] were bred in parallel with wild type mice from the same strain (129 S6/SvEv Tac, Taconic Farms, Germantown, NY). Every 3 to 4 generations *Slc26a4 (−/−)* and *Slc26a4 (+/+)* were crossed to generate heterozygotes, which were then bred to produce wild type and pendrin null litermates, which were then bred separately. Age- and sex-matched, pair-fed pendrin null and wild type mice were compared.

### Animal Conditioning

#### Treatment 1

For 7–10 days prior to sacrifice, mice were ration-fed a NaCl-replete gelled diet (24.8% Zeigler Brothers #53881300 rodent chow, 74.6% water, 0.6% agar) and supplemented with NaCl to give 0.8 meq/day NaCl or 1% NaCl [5]. *Treatment 2*: Mice were ration-fed the diet given in *Treatment 1* with candesartan cilexetil added to the diet, giving each mouse 6 mg/kg bw/day for 14 days. All studies were completed in compliance with protocols reviewed and approved by the Institutional Animal Care and Use Committee of the Atlanta Veterans Administration and the Emory University School of Medicine.

### Vessel Preparation

Male pendrin null or age-matched, male congenic wild type mice (SvEvTac, Taconic Farms) were given the NaCl-replete diet prepared as a gel or diet and candesartan and euthanized by CO_2_ asphyxiation. Thoracic aortas were dissected and prepared for contractility measurements as described previously [18], [19]. Briefly, vessels were rinsed in cold bicarbonate-buffered physiological saline solution (PSS) that contained (in mM): 118 NaCl, 4.73 KCl, 1.2 MgSO_4_, 0.026 EDTA (ethylene diamine tetraacetic acid), 1.2 NaH_2_PO4, 2.5 CaCl_2_, 25 NaHCO_3_, and 5.5 glucose bubbled with 95% O_2_/5%CO_2_ at 37°C. In studies using denuded vessels, the endothelium was removed by rubbing the ring between thumb and forefinger and confirmed by the loss of an endothelium-dependent relaxation to acetylcholine.

### Measurement of Aorta Thickness

Excised aortas were fixed in 10% buffered formalin and embedded in paraffin. Serial sections 6 µm thick were cut and stained with hematoxylin and eosin. Images were obtained on a Zeiss Axiovert 200 microscope equipped with an18.2 Color Mosaic camera (Diagnostic Instruments Inc.). In each mouse, intima and media thickness was measured at ten regions around the circumference of the vessel using a calibrated micrometer tool in ImageJ software (NIH) and the values averaged.

### Contractility measurements in the aorta

5 mm aortic rings were mounted isometrically on a hook that was attached to a Harvard Apparatus Differential Capacitor Force Transducer. Resting tension on each aorta was set at 20 mN to approximate an in vivo aortic pressure of ∼100 mm Hg. Vascular contractility was assessed by generating concentration-response curves to KCl (0–80 mM) and phenylephrine (PE, 0.1 nM to 10 µM). In other experiments vascular contractility was measured as force in response to 100 nM angiotensin II. Data were obtained using Powerlab hardware (AD Instruments, Colorado Springs, CO) and analyzed with LabChart software (AD Instruments).

At the end of the experiment, rings were cut open longitudinally and the muscle dimensions (segment length and circumference) were measured with a calibrated optical micrometer [20]. The tissue was then gently blotted and weighed (wet weight). Vessel cross-sectional area (CSA) was calculated using the relationship:



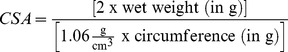
where 1.06 g/cm^3^ was used as an estimate of tissue density [20]. This approach yields values for vessel thickness that are similar to thickness measurements determined by morphometry [21].

In a separate series of studies, calcium sensitivity was examined using established techniques [22]. To Ca^2+^-deplete vessels, aortas were washed twice in the physiological saline solution (PSS) given above but CaCl_2_ was excluded from and 0.5 mM EGTA ((β-amino-ethyl ether)- N,N′ tetraacetic acid) was added to the PSS solution. Vessels were then depolarized with the addition of 50 mM KCl. Calcium (CaCl_2_) was added to the bath in 0.2 mM increments and isometric tension was monitored and analyzed using LabChart Software.

### Measurement of mRNA and protein abundance

Sex- and age- (6–8 weeks of age) matched wild type and pendrin null mice were sacrificed under anesthesia with 1–2% isofluorane in 100% O_2_ and kidneys and/or aortas were removed. Total RNA was isolated from isolated aortas and kidneys using a kit (TRizol Plus RNA Purification Kit, Invitrogen) and then DNase treated [2]. Pendrin mRNA and β-actin were quantified in the same samples using quantitative real-time polymerase chain reaction (PCR) with specific quantitative assays reported previously for mouse *Slc26a4* and β-actin mRNA [2], [5]. Quantitative real-time PCR was performed in the Quantitative Genomics Core Laboratory in the Department of Integrative Biology and Pharmacology, University of Texas, Health Science Center Houston (UTHSC).

Immunoblots of mouse kidney and aorta lysates were performed as reported previously [23]. Tissue was homogenized, dissolved in laemmli buffer and resolved by SDS polyacrylamide gel electrophoresis. Protein was electrophoretically transferred onto nitrocellulose membranes and probed with one of the antibodies described below [24]. Equal protein loading was confirmed by running a gel in parallel that was stained with Coomassie blue dye [25]. For aorta lysates, each “n” reported corresponds to tissue pooled from 2 mice. For kidney lysates, each “n” represents tissue taken from a single mouse. The primary rabbit anti-pendrin (*Slc26a4*) antibody employed in immunoblots recognizes the terminal 29 amino acids of the rat pendrin protein sequence [24], [26] and was a generous gift of Dr. Peter Aronson. The rabbit, anti-human N-terminal myosin light chain 20 antibody was obtained from Cell Signaling Technology (#3672). The rabbit, anti-human N-terminal smooth muscle α actin antibody was purchased from Thermo Scientific (RB-9010) while the rabbit, anti-mouse C-terminal Cdk4 antibody was purchased from Santa Cruz (sc 260). Myosin heavy chain (MHC SM1 and SM2) antibodies, characterized previously [27], [28], were a generous gift of Dr. Anne F. Martin. Immunolabeling was detected with horseradish peroxidase-conjugated goat anti-rabbit secondary antibody (Upstate Biotechnology Inc., Lake Placid, NY) using an enhanced chemiluminescence system (Amersham Biosciences, Little Chalfont, UK). Band density was quantified using Quantity One Image software (Biorad, Hercules, CA) and compared between groups.

### MLC 20 phosphorylation

MLC20 phosphorylation was examined by 2-dimensional electrophoresis as previously described [29]. Control and phenylephrine treated aortae were rapidly frozen at predetermined times and then transferred to 10% trichloroacetic acid in acetone (−70°C). After being stored for at least 12 h at −70°C, samples were slowly brought to room temperature and then washed extensively with acetone and dried. Tissue was homogenized in a buffer containing 1% SDS, 10% glycerol, 20 mM dithiothreitol, and 5 mg/ml bovine serum albumin. Tissue homogenates were separated by two-dimensional electrophoresis at pH 4.5–5.4 with 15% SDS-polyacrylamide gels and then silver stained and quantified by densitometry. Percent phosphorylated MLC20 was determined by the relationship: (P1+P2)/(U+P1+P2), where U is the unphosphorylated light chain spot and P1 & P2 are, respectively, the singly and doubly phosphorylated light chains.

### Catecholamine levels

Mice were placed in metabolic cages 48 hours prior to sacrifice. Urine was collected continuously during the 24 hrs prior to sacrifice under oil in 0.1 N HCl. After collection, urine was centrifuged and the supernatant collected. Urinary catecholamine concentration was measured in duplicate, 50-µl aliquots of mouse urine using alumina extraction followed by HPLC with electrochemical detection. The method employs automated in-line cationic trace enrichment [30]–[32] and was adapted to a 96-well microplate format using Multiscreen Solvinert Deep Well filter plates (Millipore Corporation, Billerica, MA, Cat No. MDRL NO4 10). An internal standard (dihydroxybenzylamine) was used to control for losses during extraction and the system was calibrated using norepinephrine and epinephrine standards from Sigma (St. Louis, Mo) (N3146-1VL and E1016-1VL respectively). Results are expressed as mass of free base excreted in the urine/24 hr. The overall coefficient of variation between duplicates was 5.7% for norepinephrine and 8.4% for epinephrine.

### Statistics

When two groups were compared, an unpaired Student's t test was employed. Multiple groups were compared with ANOVA with a Bonferoni post test. Data are expressed as the mean ± SE. A P<0.05 indicates statistical significance.

## Results

### Pendrin gene ablation increases aorta contractile force per cross sectional area without affecting contractile sensitivity

**Figure 1:**
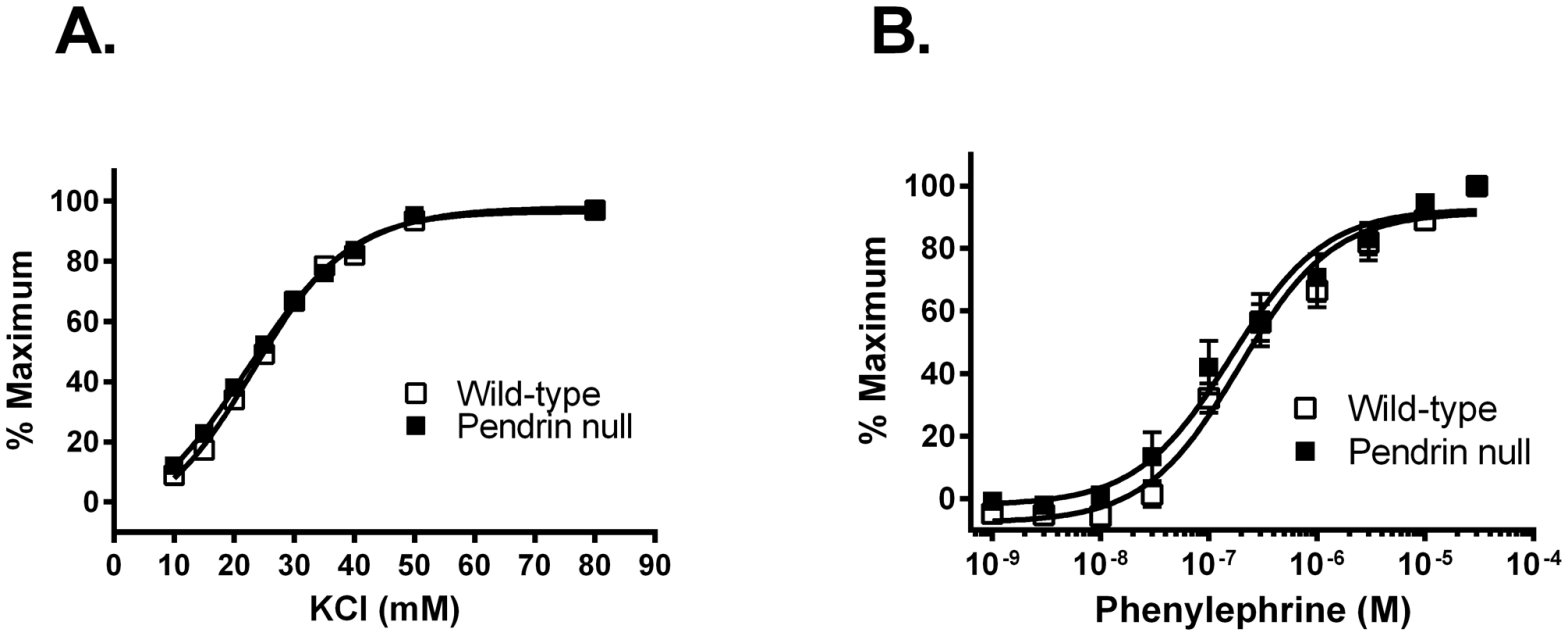
Pendrin gene ablation does not affect aortic contractile sensitivity to KCl (A) and phenylephrine (PE;B). Aortic rings from wild type and pendrin null mice were isometrically mounted and concentration response curves were generated to KCl and phenylephrine. Panels A and B show the dose-response to KCl and phenylephrine normalized to maximum force (n = 8–9).

Following 7 days of the NaCl-replete diet employed in this study, we observed previously that mean arterial pressure measured by telemetry is lower in pendrin null than in wild type mice [9], [16]. Further experiments asked if the fall in blood pressure observed with pendrin gene ablation occurs in tandem with reduced vascular reactivity. To examine the effect of pendrin gene ablation on vascular contractility, isometric force was measured in thoracic aortic rings from pendrin null and wild type mice in response to the cumulative addition of the α adrenoceptor agonist, phenylephrine, (10^−10^ to 10^−5^ M) or the depolarizing agent, KCl (0–80 mM). Contractile sensitivity was not affected by pendrin gene ablation since the EC_50_ values for phenylephrine (wild type, 280±12 nM; pendrin null, 261±11 nM, P = NS) and KCl (wild type, 23.2±1.2 mM; pendrin null 21.3±1.2 mM, P = NS) were not different in the mutant and the wild type aorta (Figures 1A and 1B).

**Figure 2:**
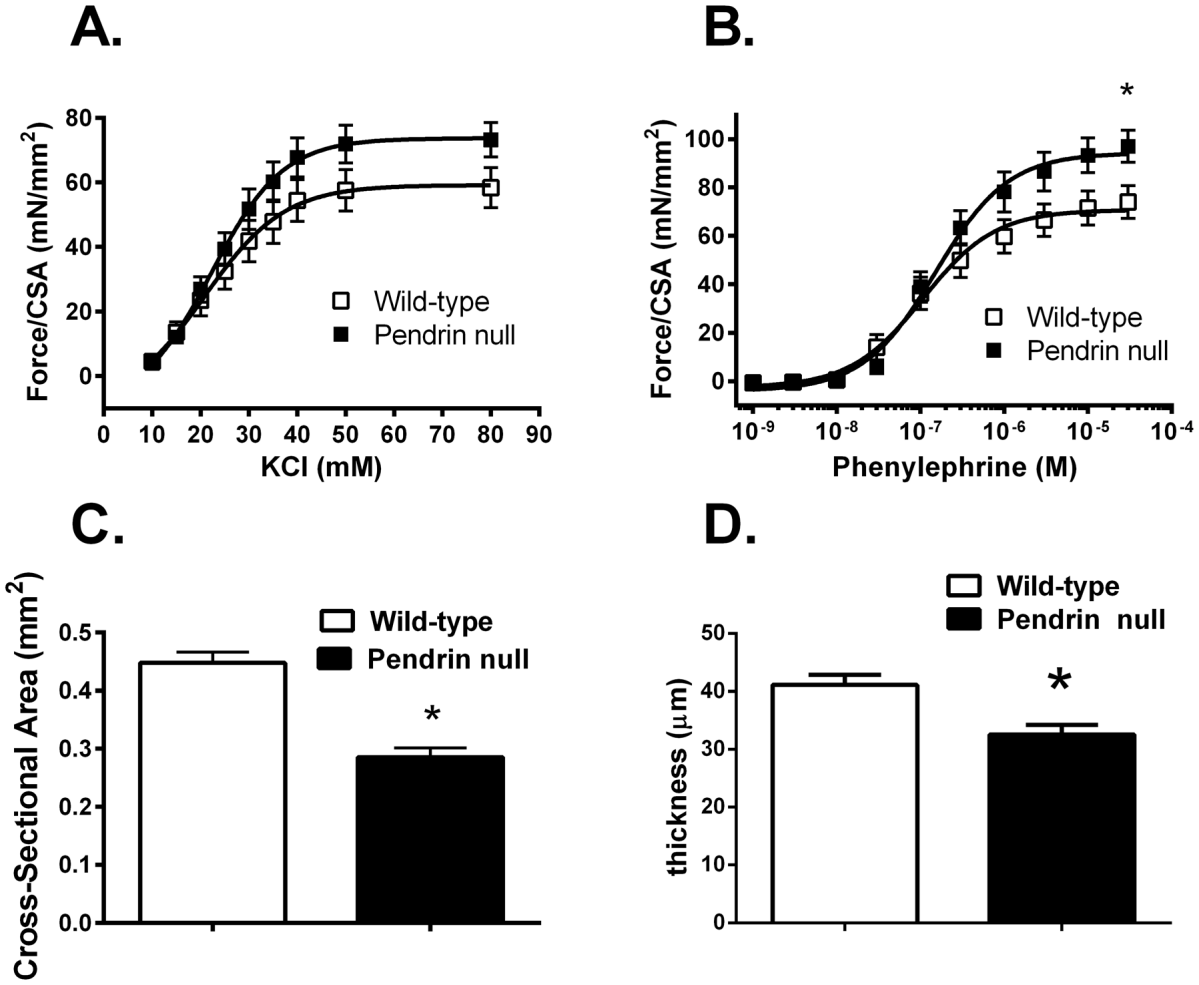
Pendrin gene ablation increases force/cross-sectional area and decreases thickness of the aorta. Aortic rings from wild type and pendrin null mice were isometrically mounted. Maximum force generated/cross sectional area was measured in response to KCl (Panel A) and phenylephrine (PE, Panel B) (n = 5–9). Cross sectional area (CSA, Panel C) was measured in the same vessels using the relationship 2 x wet weight/circumference. Aorta thickness (Panel D) was measured in transverse sections using a calilbrated micrometer in pendrin null (n = 4) and wild type mice (n = 5). The body weights for aorta thickness measurements were 30.0±0.8 g in wild type versus 26.8±1.1 g in pendrin null mice. *p<0.05.

Further experiments examined force development normalized to cross sectional area in thoracic aortas from pendrin null and wild type mice. In response to KCl, force generation/cross sectional area trended higher in the pendrin null relative to the wild type aorta, although differences did not reach statistical significance (Figure 2A). However, in response to phenylephrine (PE), force/cross-sectional area was increased in the pendrin null relative to the wild type aorta (Figure 2B).

Because pendrin gene ablation increased force when normalized to cross sectional area, we compared cross sectional area in the thoracic aorta from mice in each group. As shown, cross sectional area was lower in aortas from pendrin null relative to wild type mice (Figure 2C). Similarly, aorta intima and media thickness quantified by planar morphometry was lower in pendrin null than in wild type mice (Figure 2D), which is consistent with the 11% lower body weight observed in the pendrin null relative to wild type mice. We conclude that the enhanced force/per cross sectional area observed in the pendrin null aorta was primarily due to reduced cross-sectional area rather than increased force/vessel.

### Pendrin expression is very low in mouse aorta

Intracellular Cl^−^ is maintained above equilibrium in smooth muscle partly through robust Cl^−^/HCO_3_^−^ exchange, which may alter smooth muscle tone [33]. We therefore reasoned that pendrin-mediated Cl^−^/HCO_3_^−^ exchange within aorta smooth muscle may alter contractile force. As such, we asked if the increased contractile force observed in the pendrin null aorta is the due to the absence of pendrin-mediated Cl^−^/HCO_3_^−^ exchange in aorta smooth muscle or if it occurs through an indirect and possible systemic effect of pendrin gene ablation. Thus *Slc26a4* mRNA and pendrin protein were quantified in mouse aorta. Pendrin mRNA and protein were also quantified in kidney as a positive control. The percent *Slc26a4* relative to β actin mRNA in kidney tissue reported in Table 1 is very similar to values reported previously in the renal cortex of mice studied under the same treatment conditions [2]. However, both the ratio of *Slc26a4* to β actin mRNA and the ratio of *Slc26a4* mRNA to total RNA were more than 2 orders of magnitude lower in aorta than in whole kidney (Table 1). Thus the level of *Slc26a4* mRNA expression is very low in mouse aorta.

**Table 1:** *Slc26a4* mRNA levels in wild-type aorta and kidneys.

Sample	*Slc26a4* template molecules/100 ng total RNA	β-actin template molecules/100 ng total RNA	*Slc26a4*/β-actin mRNA %
*Aorta*			
Wild type	327±101 (n = 7)	2,962±428×10^3^ (n = 7)	0.0134±0.003 (n = 7)
*Kidney cortex*			
Wild type	73,135 (n = 2)	2,083×10^3^ (n = 2)	3.243 (n = 2)

**Figure 3:**
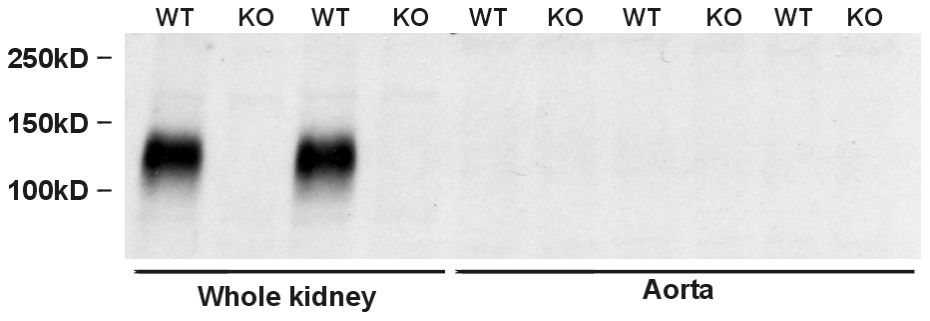
Pendrin protein expression is not detectable in mouse aorta. Pendrin immunoreactivity was explored by immunoblot of lysates from kidney and aorta of wild type (WT) and pendrin null (KO) mice. Each lane was loaded with 20 µg protein.

Further studies explored whether pendrin protein is expressed in mouse aorta. Pendrin protein was not detected in aorta lysates from either wild type or pendrin null mice, despite robust pendrin abundance detected in kidney lysates from wild type mice run in parallel (Figure 3). We conclude that pendrin protein and mRNA are either extremely low or undetectable in mouse aorta. Therefore, the change in contractile force observed in the pendrin null aorta occurs through an indirect effect of pendrin gene ablation, such as through changes in the production of or the sensitivity to a vasoactive hormone.

### Changes in contractile force observed in pendrin null mice are not from changes in sensitivity to nitric oxide or Ca^2+^ or from changes in catecholamine production

**Figure 4:**
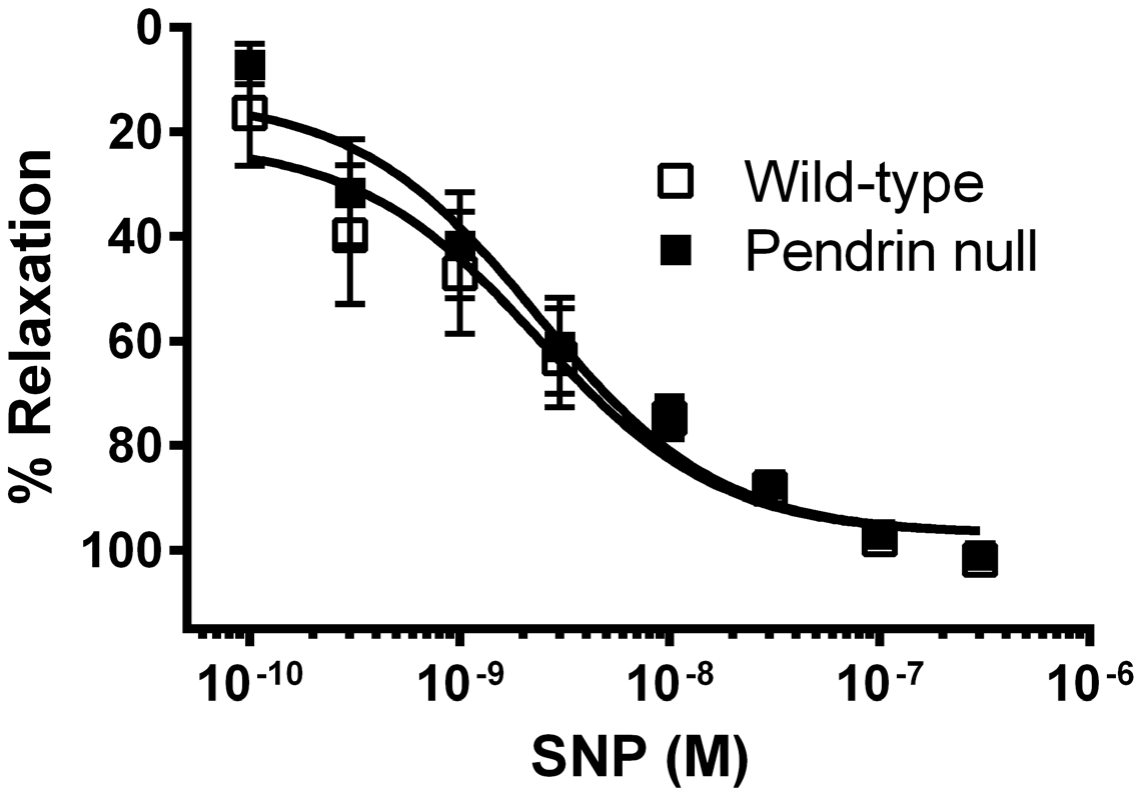
Pendrin gene ablation does not affect nitric oxide-dependent smooth muscle relaxation. Aortic rings from wild type and pendrin null mice were preconstricted with 30 nM phenylephrine. Relaxation in response to sodium nitroprusside (SNP, 0.1 to 300 nM)) was then determined. Data are expressed as the percent relaxation in response to the incremental addition of SNP (n = 5).

Further experiments explored how pendrin gene ablation alters force generation in the thoracic aorta. Since nitric oxide produced by the endothelium modulates vascular contractility by relaxing vascular smooth muscle [34], we examined relaxation responses to an NO donor (sodium nitroprusside, SNP) in aortas from wild-type and pendrin null mice. In preconstricted vessels, the percent relaxation in response to SNP was similar in wild type and in pendrin null mice (Figure 4). Since vascular relaxation in response to NO was similar in aortas from pendrin null and wild type mice and since pendrin gene ablation increased contractile force/cross sectional area in both intact and denuded aortas (not shown), changes in force/cross sectional area that follow pendrin gene ablation do not occur from changes in NO sensitivity. Therefore, genetic disruption of the gene encoding pendrin does not affect nitric oxide-mediated relaxation.

**Figure 5:**
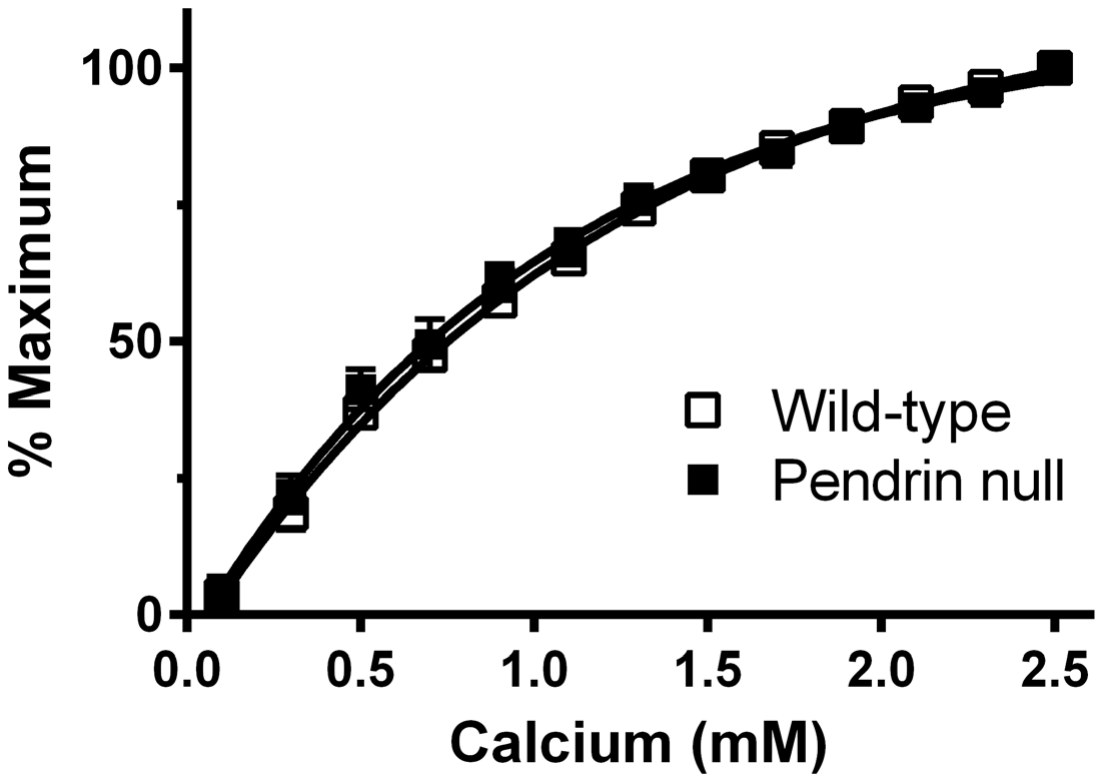
Pendrin gene ablation does not increase aortic contractile force through changes in Ca^2+^ sensitivity. Rings from wild type and pendrin null mice were Ca^2+^ depleted by incubating in the presence of the Ca^2+^ chelator, EGTA, and then depolarized with 50 mM KCl. Vascular contractility was measured following the addition of calcium to the bath in increments of 0.2 mM [22], n = 5–6.

Further experiments explored whether pendrin gene ablation decreases aorta smooth muscle contractility through changes in Ca^2+^ sensitivity [35]. Therefore, we examined the calcium sensitivity of contractile force (Figure 5) in aortas from pendrin null and wild type mice. To do so, aortic rings were Ca^2+^-depleted and then depolarized. Following the step-wise addition of Ca^2+^ [22], vascular contractility was similar in aortic rings from pendrin null and wild type mice. We conclude that the increased contractile force observed with pendrin gene ablation does not occur from changes in smooth muscle Ca^2+^ sensitivity.

**Figure 6:**
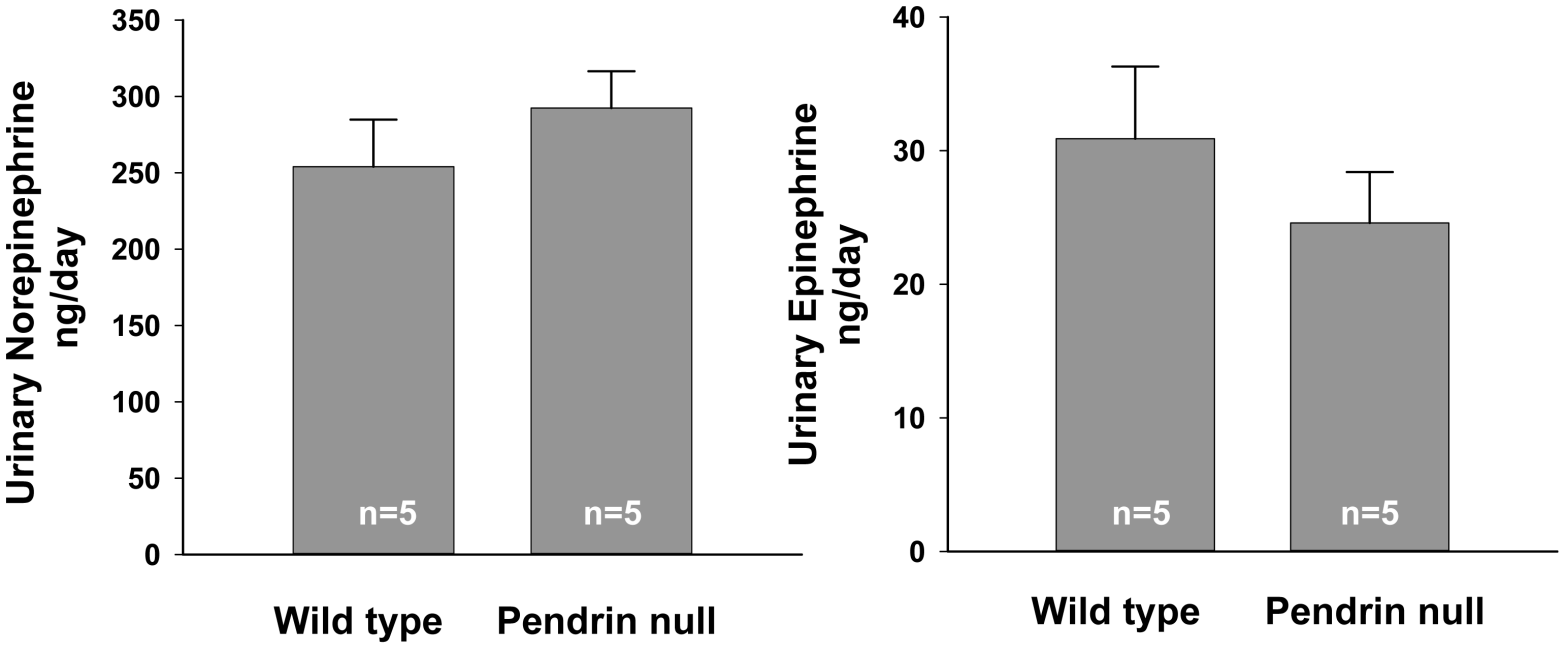
Pendrin gene ablation does not affect steady state catecholamine production. 24 hour urinary epinephrine and norepinephrine excretion of pendrin null and wild type mice are shown.

Since chronic norepinephrine administration reduces the maximal force of contraction [36], we asked if catecholamine release differs in pendrin null mice and wild type mice. Thus, urinary epinephrine and norepinephrine were measured in wild-type and pendrin null mice. As shown (Figure 6), 24 hr urinary excretion of epinephrine and norepinephrine was similar in wild-type and pendrin knockout mice. Thus, the increase in aortic contractility/cross sectional area observed with pendrin gene ablation cannot be explained by changes in basal levels of catecholamine production.

### Pendrin null mice have increased angiotensin II receptor signaling, which augments smooth muscle contractile force/cross sectional area

**Figure 7:**
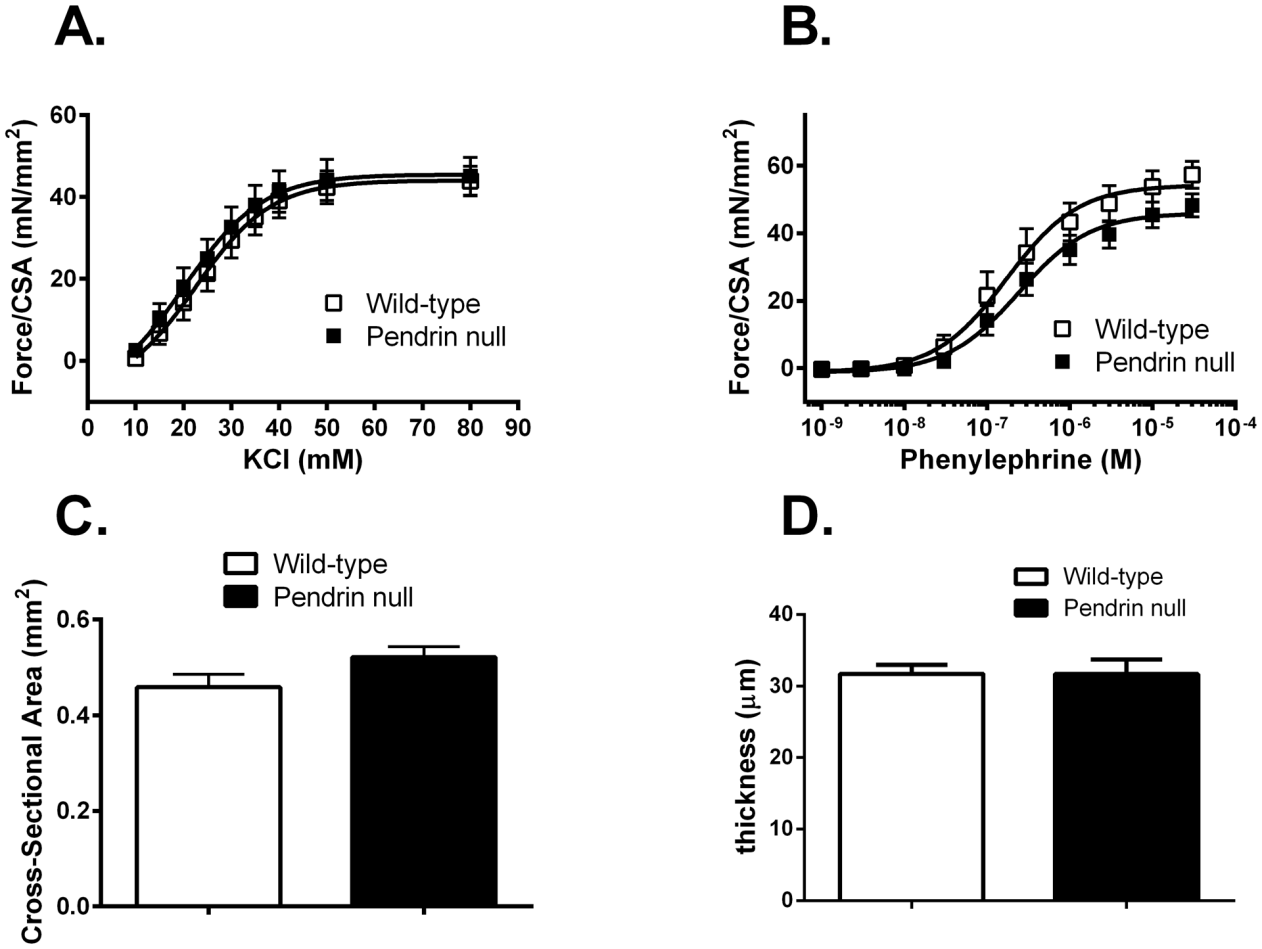
Inhibition of the angiotensin type 1 receptor equalizes aortic contractile force and cross sectional area in pendrin null and wild type mice. Aortic rings from candesartan-treated wild type and pendrin null mice were isometrically mounted and maximum force generated/cross sectional area was measured in response to the cumulative addition of KCl (Panel A) and PE (Panel B) (n = 5–6). Cross sectional area of the aorta wall was measured in each of the vessels studied (Panel C). Aorta thickness (intima and media, Panel D) was measured by morphometry in separate candesartan-treated wild type (n = 6) and pendrin null (n = 6) mice. Body weights for the aorta thickness measurements were the following: wild type 24.4±0.9 g versus 21.4±1.0 g for pendrin null mice.

We reasoned that angiotensin II-depending signaling might mediate the observed change in cross sectional area observed with pendrin gene ablation since this peptide hormone induces vascular remodeling [37]. Therefore, we asked if the increased force/area observed in pendrin null mice is angiotensin II-dependent. To answer this question, we explored the effect of 2 weeks of angiotensin type 1 receptor blocker treatment (candesartan) on maximal contractile force. As shown (Figure 7 A & B), the maximal force of contraction/cross sectional area in response to PE and KCl were similar in thoracic aortas from candesartan-treated pendrin null and wild type mice. Moreover, aorta cross sectional area and thickness of the aorta wall were similar in the mutant and wild type mice following candesartan treatment (Figure 7 C & D), despite the 12% lower body weight observed in the pendrin null relative to the wild type mice. Thus angiotensin type 1 receptor blockade eliminated differences between pendrin null and wild type mice in aorta contractile force normalized to cross sectional area. Candesartan normalized force/CSA in the mutant and wild type mice by increasing the relative cross sectional area of the pendrin null relative to the wild type aorta rather than by changing force per vessel.

### Aortas from pendrin null mice have increased contractile protein abundance

Because pendrin gene ablation changed aortic wall thickness through an angiotensin type 1 receptor-dependent mechanism, further experiments characterized the angiotensin-dependent vascular remodeling that follows pendrin gene ablation. We asked if the increased contractile force observed in the pendrin null aorta is accompanied by a change in the pattern of contractile protein abundance and if differences in contractile protein abundance are eliminated with candesartan treatment. Thus, we explored the effect of pendrin gene ablation on the abundance of actin and myosin and the abundance of a ubiquitously expressed “housekeeping” protein (cdk4).

**Figure 8:**
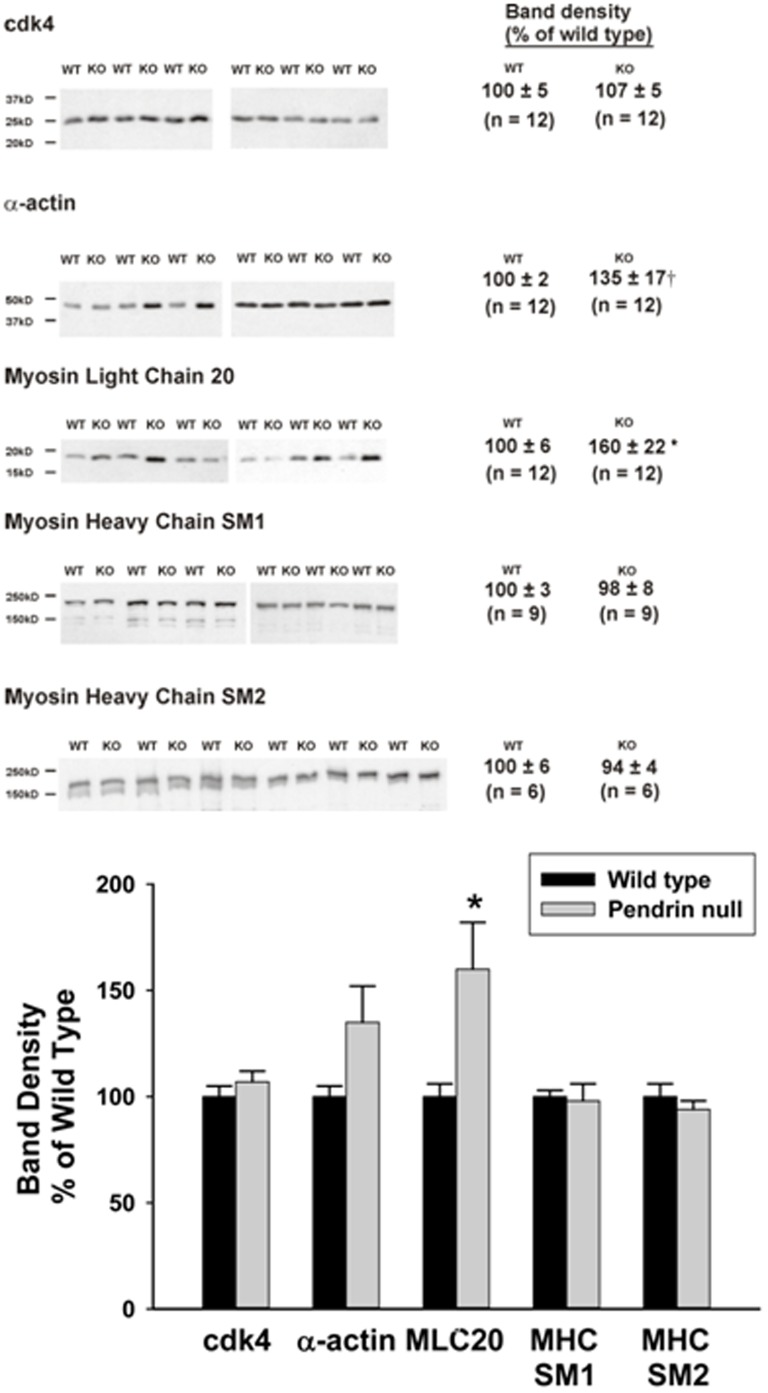
Pendrin gene ablation changes the pattern of contractile protein expression. Contractile protein abundance was quantified by immunoblot of aorta lysates from pendrin null and wild type mice. Representative western blots (Top Panel) and band densities normalized to wild type mice (Bottom Panel) are shown. *p<0.05; ^†^p = 0.063

**Figure 9:**
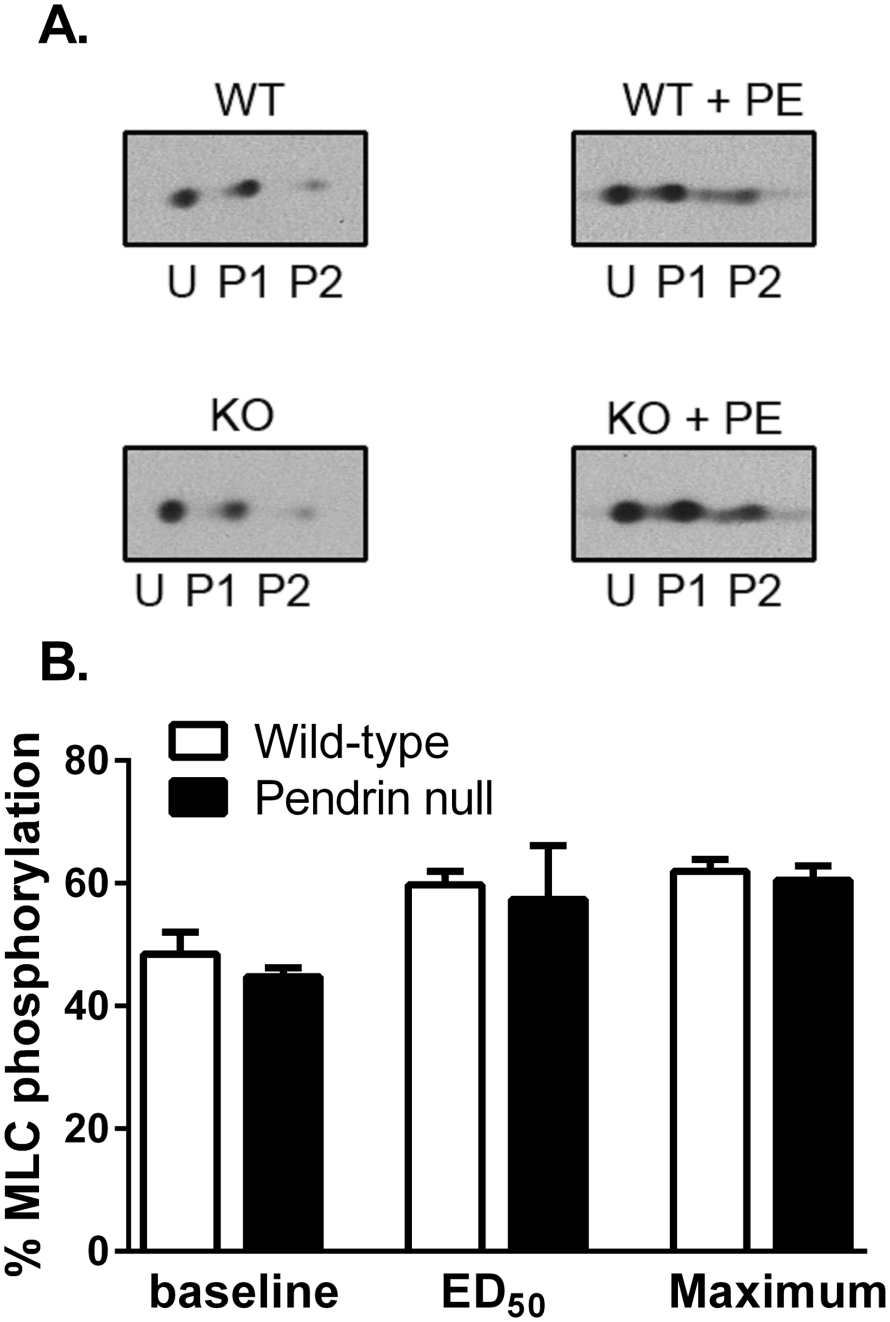
Pendrin gene ablation does not change the percentage of myosin light chain phosphorylation in the mouse aorta. Aortas were isometrically mounted and phosphorylation was determined under either baseline conditions or following stimulation with phenylephrine at the ED_50_ concentration (0.3 µM) or at a concentration giving maximal stimulation (10 µM). The percentage of MLC20 phosphorylation was determined using 2-D electrophoresis. U represents the unphosphorylated light chain spot, whereas P1 & P2 are, respectively, the singly and doubly phosphorylated light chains. N = 4 in each group.

Since the relative abundance of α actin and smooth muscle myosin heavy chain isoforms can significantly impact force development [21], [38], [39], we quantified the abundance of each myosin heavy chain isoform (MHC SM1 and MHC SM2) as well as α actin. As shown (Figure 8), the abundance of α actin, SM1 and SM2 and the ratio of SM1/SM2 (0.973±0.04, n = 6, versus 1.01±0.07, n = 6, respectively, P = NS) were similar in aortas from wild type and pendrin null mice. However, since the primary signaling event that initiates cross-bridge cycling of smooth muscle is the phosphorylation of the 20 kDa myosin regulatory chain (MLC20) [40], [41], we measured total and phosphorylated MLC20 abundance in aortas from wild type and pendrin null mice (Figure 8). Total MLC20 abundance was 60% higher in pendrin null than in wild type mice (Figure 9), although the percent phosphorylated MLC20 was similar in aortas from both wild type and mutant mice under basal conditions and following the application of phenylephrine either at its EC50 or at its maximal stimulatory concentration (Figure 9). However, since total MLC abundance was 60% higher in aortas from pendrin null mice, phosphorylated MLC abundance is also higher in aortas from pendrin null relative to wild type mice. We conclude that total and phosphorylated myosin light chain abundance is upregulated in pendrin null mice.

**Figure 10:**
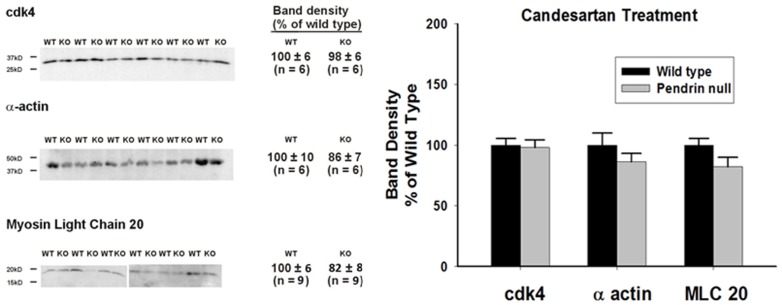
Angiotensin receptor type 1 inhibition eliminates the increase in MLC20 protein abundance observed in aortas from pendrin null mice. Pendrin null and wild type mice were given candesartan for 14 days and then sacrificed. Contractile protein abundance (α actin and MLC 20) and a “housekeeping gene” (cdk4) were quantified by immunoblot in lysates of aortas taken from mice in each group. Representative immunoblots (Left Panel) and band density normalized to wild type mice (Right Panel) are shown.

Because angiotensin type 1 receptor blocker (candesartan) application in vivo eliminated the increased contractile force observed in pendrin null mice, we asked if candesartan changed the relative abundance of contractile proteins in the pendrin null aorta. As shown (Figure 10), following candesartan treatment, α actin and MLC20 abundance were similar in aortas from pendrin null and wild type mice. We conclude that 1) pendrin gene ablation increases the abundance of thoracic aorta contractile proteins, such as MLC20, and 2) blockade of angiotensin receptor type 1-mediated signaling modulates changes in contractile protein abundance observed with pendrin gene ablation.

### Pendrin gene ablation increases maximal aorta contractile force in response to angiotensin II

**Figure 11:**
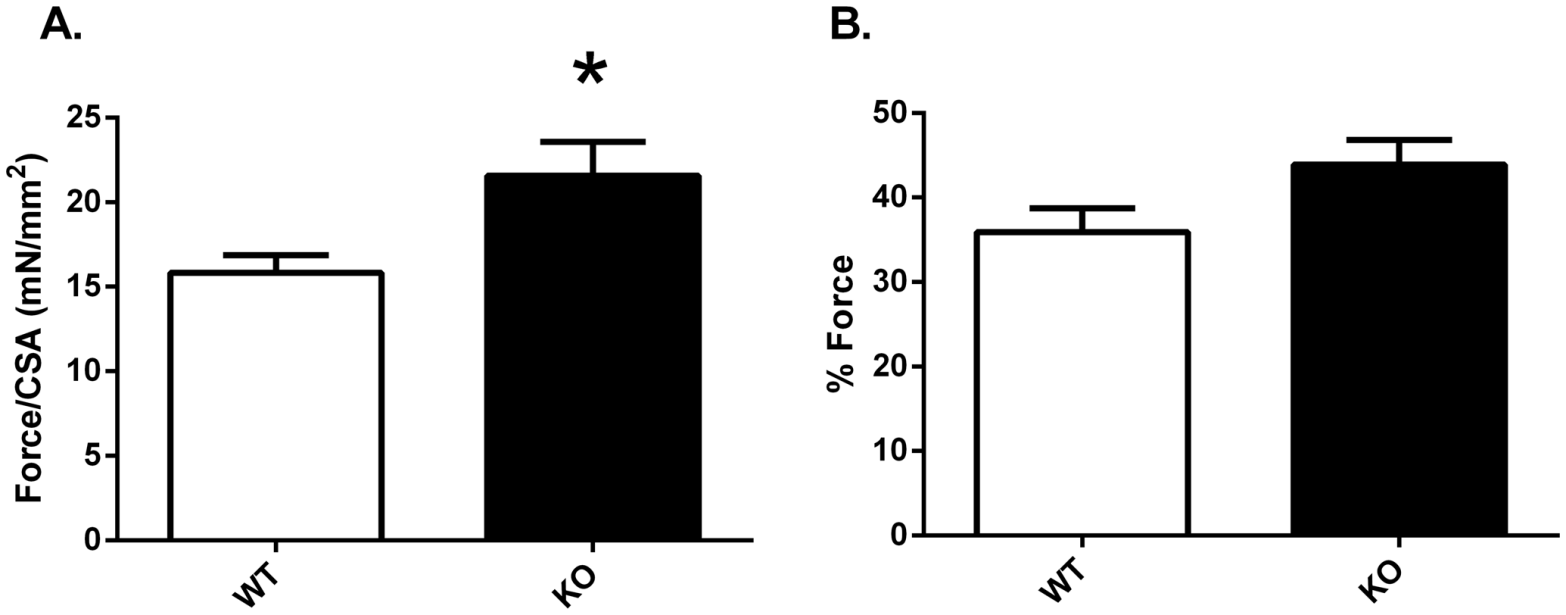
Force/CSA in response to Angiotensin II in isolated mouse thoracic aorta from wild type and pendrin null mice. Force/CSA was measured in response to angiotensin II (100 nM) in thoracic aortas from pendrin null (KO) and wild type (WT) mice (Panel A). Panel B shows Force/CSA in response to 100 nM angiotensin II when expressed as the percentage of force/CSA observed in response to phenylephrine (10 µM). n = 7 in each group. *p<0.05.

Further experiments examined the effect of pendrin gene ablation on aorta contractile force in response to 100 nM angiotensin II, a concentration shown to generate maximal force in mouse thoracic aorta [42], [43]. As shown (Figure 11A), in response to angiotensin II, force/CSA was greater in the pendrin null than in the wild type thoracic aorta. To determine if the angiotensin II response was exaggerated in the pendrin null aorta, the force response to angiotensin II was expressed relative to the force response to PE (% Force). As shown (Figure 11B), % Force was similar in aortas from these two groups. We conclude that pendrin gene ablation increases force/CSA generated in response to both PE and to angiotensin II.

## Discussion

Following 7 days of the NaCl-replete, gelled diet employed in this study, the present and previous studies have shown that mean arterial blood pressure is 5 to 9 mm Hg lower in pendrin null relative to wild type mice when measured by telemetry [9], [16]. Whereas we observed mean arterial blood pressures of 116 to 122 mm Hg in wild type mice, MAP was in the range of 113 to 116 mm Hg in pendrin null mice [9] [16].

Whether the fall in blood pressure observed with pendrin gene ablation is accompanied by changes in vascular tone has been unexplored. We hypothesized that since blood pressure is reduced in pendrin null mice, vascular reactivity and the contractile response should be attenuated in the mutant mice [44], [45]. Our results indicate that pendrin gene ablation increases contractile force normalized to cross sectional area in response to phenylephrine and angiotensin II. However, the sensitivity to contractile agents such as phenylephrine or KCl is unaffected. Since pendrin mRNA and protein are not detectable in conduit vessels (aorta), pendrin gene ablation alters aorta contractility through an indirect and possibly systemic effect of pendrin gene ablation.

Since thyroid hormone, aldosterone and cortisol levels are the same in pendrin null and wild type mice under the conditions of the present study [17], [24], the differences in contractility observed in the present study cannot be explained by changes in the production of these hormones. However, under the treatment conditions employed in the present study (i.e. a NaCl-replete diet given as a gel), circulating renin concentration is two-fold higher in pendrin null relative than in wild type mice (wild type, 0.2±0.01versus 0.4±0.07 µg ANG I. ml^−1^ h^−1^, in pendrin null mice) [8], [9]. Renin production is stimulated in the pendrin null mice, at least in part, from the reduced circulating volume [46] and from the lower blood pressure [9], [16] observed in these mutant mice. This increase in renin production should stimulate angiotensin II release. Increased circulating angiotensin II levels and/or changes in angiotensin type 1 receptor expression likely contribute to the increased vascular force observed in this study.

Angiotensin II application in vitro acts through the angiotensin type 1a receptor to increase contractile force in the rodent aorta [15]. The present study shows that pendrin gene ablation enhances the force/CSA generated in response to angiotensin II when applied to aortas ex vivo. The increased contractile force observed in the pendrin null aorta in response to angiotensin II might occur through changes in angiotensin type 1 receptor expression [15]. Alternatively, it may occur through changes in contractile protein expression, independent of angiotensin type 1 receptor changes, thereby making the tissue more angiotensin II sensitive.

Angiotensin II increases vascular contractility by raising vascular smooth muscle contractile protein abundance [47]. These changes in contractile protein abundance occur in tandem with an increased capacity of the remodeled pendrin null aorta to generate force. In particular, we observed an increase in MLC20 in the pendrin null aorta, which may augment the agonist-induced contractility observed in this vessel. Whether chronic elevations in vascular smooth muscle MLC abundance in vivo result in sustained increases in contractile force remains to be determined [35]. Moreover, we cannot exclude the possibility that pendrin gene ablation alters the expression of additional proteins that were not examined in this study.

Pendrin abundance in the apical plasma membrane of renal intercalated cells falls when mice are given a NaCl-replete diet, a condition during which renin, angiotensin II and aldosterone production decline. Conversely, renal apical plasma membrane pendrin abundance increases during treatment models that stimulate renin, angiotensin and aldosterone release, such as a NaCl-deficient diet [4]. In kidney, angiotensin II acts through the angiotensin type 1 receptor to upregulate pendrin, thereby increasing blood pressure [7]. Pendrin gene ablation therefore reduces blood pressure much more during treatment conditions in which renin, angiotensin or aldosterone release is stimulated than following a NaCl-replete diet, where renin, angiotensin II and aldosterone production are suppressed [4]–[7], [9]. Angiotensin II-dependent vascular remodeling may provide a mechanism to blunt the fall in blood pressure produced with pendrin gene ablation.

In summary, our results indicate that in mouse aorta pendrin gene ablation increases the force of contraction per cross sectional area. The increased force observed in the pendrin null aorta that is generated in response to PE and angiotensin II is not a direct result of changes in pendrin expression in this vascular tissue, but results instead from an indirect and possibly systemic effect of pendrin gene ablation that occurs through angiotensin II-depending signaling. Ablation of the gene encoding pendrin increases contractile force/CSA in mouse aorta in response to angiotensin II and PE through a mechanism dependent on the angiotensin type 1 receptor. This angiotensin II-dependent signaling pathway observed in vascular smooth muscle may help maintain blood pressure following pendrin gene ablation.
